# Multi-Pass Adaptive Voting for Nuclei Detection in Histopathological Images

**DOI:** 10.1038/srep33985

**Published:** 2016-10-03

**Authors:** Cheng Lu, Hongming Xu, Jun Xu, Hannah Gilmore, Mrinal Mandal, Anant Madabhushi

**Affiliations:** 1 College of Computer Science, Shaanxi Normal University, Xi’an, Shaanxi Province, 710119, China; 2 Department of Biomedical Engineering, Case Western Reserve University, Cleveland, OH, 44106-7207, USA; 3 Department of Electrical and Computer Engineering, University of Alberta, Edmonton, Alberta, T6G 2V4, Canada; 4 Jiangsu Key Laboratory of Big Data Analysis Technique, Nanjing University of Information Science and Technology, Nanjing, 210044, China; 5 Department of Pathology-Anatomic, University Hospitals Case Medial Center, Case Western Reserve University, Cleveland, OH, 44106-7207, USA

## Abstract

Nuclei detection is often a critical initial step in the development of computer aided diagnosis and prognosis schemes in the context of digital pathology images. While over the last few years, a number of nuclei detection methods have been proposed, most of these approaches make idealistic assumptions about the staining quality of the tissue. In this paper, we present a new Multi-Pass Adaptive Voting (MPAV) for nuclei detection which is specifically geared towards images with poor quality staining and noise on account of tissue preparation artifacts. The MPAV utilizes the symmetric property of nuclear boundary and adaptively selects gradient from edge fragments to perform voting for a potential nucleus location. The MPAV was evaluated in three cohorts with different staining methods: Hematoxylin & Eosin, CD31 & Hematoxylin, and Ki-67 and where most of the nuclei were unevenly and imprecisely stained. Across a total of 47 images and nearly 17,700 manually labeled nuclei serving as the ground truth, MPAV was able to achieve a superior performance, with an area under the precision-recall curve (AUC) of 0.73. Additionally, MPAV also outperformed three state-of-the-art nuclei detection methods, a single pass voting method, a multi-pass voting method, and a deep learning based method.

Nuclei detection is often a critical initial step in the development of computer aided diagnosis and automated tissue grading schemes in the context of digital pathology images^1^,^2^,^3^,^4^,^5^,^6^,^7^,^8^,^9^,^10^,^11^,^12^. However, nuclei detection is a challenging task in images with poor staining and noise. In breast cancer diagnosis, the Nottingham Histologic Score system is highly correlated to the appearance of cancer nuclei^13^. In melanomas, the quantitative assessment of melanocytes within the epidermis is an important cue for disease presence^14^,^15^. It is clear therefore that features pertaining to nuclear shape and spatial distribution of cell nuclei have important diagnostic value^12^. However, manually identifying the location and extent of melanocyte invasion or breast cancer nuclei can be subjective and time consuming. With recent interest in developing automated and computerized cancer diagnosis and grading schemes^7^,^16^,^17^,^18^, there is a need for improved methods for nuclei detection and counting.

With recent advent of digital pathology, a number of groups have recently proposed approaches for nuclei detection^1^,^14^,^15^,^19^,^20^,^21^,^22^,^23^,^24^,^25^,^26^. Most of these approaches make assumptions about the staining quality of the tissue and the resulting image quality and consequently may yield sub-optimal detection performance if the tissue preparation and staining is less than ideal. Due to the uneven absorption of the staining dyes by the tissue, variations in staining procedures adopted in various labs, variations in the exposure time for stain absorption by the tissue, differences in the quality of biochemical tissue staining and resulting appearance of the slides can be substantial. This can adversely affect the appearance of the tissue and the associated histologic primitives such as nuclei. For instance, over-staining of hematoxylin can cause adjacent nuclei to appear to be clumped together, over-staining with eosin can cause the nuclei to blend in with the stroma and result in under-emphasis of the nuclear boundaries. Uneven and imprecise staining can also cause hollow cores within the centers of individual nuclei. An example is shown in Fig. 1(b). Unlike the image shown in Fig. 1(a) where all nuclei are uniformly stained and have a consistent appearance, most nuclei in Fig. 1(b) appear to have an ill-defined nuclear contour, with a hollow interior. While a number of gradient based approaches for nuclear segmentation have been proposed, e.g., active contour^19^ and level set schemes^21^, these approaches typically require model initialization. However, because gradient based approaches rely on the presence of strong edges for the model to latch onto, they will invariably yield worse performance on poorly stained images where the nuclei are either not uniformly stained or there are cavities within the nuclei. While deep learning (DL) based approaches^13^ have the ability to learn discriminative features, the heterogeneous appearance of nuclei in different cohorts and often the availability of only a small number of representative training samples may hinder these supervised learning based approaches. Thus, it is necessary to develop improved, efficient methods to identify individual nuclei on pathology images.

Apart from the gradient based approaches, another class of nuclear detection approaches is the voting family of methods. By utilizing the inherent symmetric property of individual nuclei as a cue, voting based methods aggregate the votes provided by each pixel on the nuclear contour. Since the nuclear contour is generally symmetric, the center region is expected to have a higher value than other regions. While the hollow interior and ill-defined nuclear contour may affect other methods, the voting based methods^22^,^24^,^27^ are typically able to handle the limitations of other families of nuclei detection methods due to their ability to ignore the heterogeneous appearance of nuclei and infer the center of nuclei from broken edge fragments.

In this paper, we present a Multi-Pass Adaptive Voting (MPAV) method that will adaptively select/modify the gradient information on nuclear contours. Unlike existing voting based methods, pixels that will join in the voting procedure will be adaptively selected and refined. This could potentially help alleviate the issue of incorrect voting, resulting in more accurate detection results. In MPAV, we assume that the shape of the objects of interest, i.e., nuclei, is convex. This is generally speaking a valid assumption since nuclei typically have elliptical shapes, which are convex.

## Previous Related Work and Novel Contributions

Table 1 enumerates some recent techniques for nuclei detection. Most approaches typically tend to use image derived cues, such as color/intensity^25^,^28^,^29^,^30^,^31^, edges^19^,^21^,^24^,^32^,^33^,^34^, texture^35^, self learned features^13^,^36^, and symmetry^22^,^24^,^27^,^37^.

The color and texture-based methods require consistent color/texture appearance for the individual nuclei in order to work optimally. The method presented in ref. 31 applied the Laplacian of Gaussian (LoG) filter to detect the initial seed points representing nuclei. However, due to the uneven distribution of nuclear stain, the response of LoG filter may not reflect the true nuclear center. Filipczuk *et al*. applied circular Hough transform to detect the nuclear center^34^, however the circular Hough transform assumes that the shape of the underlying region of interest can be represented by a parametric function, i.e., circle or ellipse. In poorly stained tissue images, the circular Hough transform is likely to fail due to the great variations in appearance of nuclear edges and the presence of clusters of edge fragments.

Recently, there has been substantial interest in developing and employing DL based methods for nuclei detection in histology images^13^,^36^. The DL methods are supervised classification methods that typically employ multiple layers of neural networks for object detection and recognition. They can be easily extended and employed for multiple different classification tasks. Recently a number of DL based approaches have been proposed for image analysis and classification applications in digital pathology^13^,^36^. For instance, Xu *et al*. proposed a stacked sparse autoencoder (SSAE) to detect nuclei in breast cancer tissue images. They showed that the DL scheme was able to outperform hand-crafted features on multi-site/stain histology images. However, DL methods required a large number of dedicated training samples since the learning process requires a large number of parameters to be learned. These approaches therefore tend to be heavily biased and sensitive to the choice of the training set.

**Figure 2:**
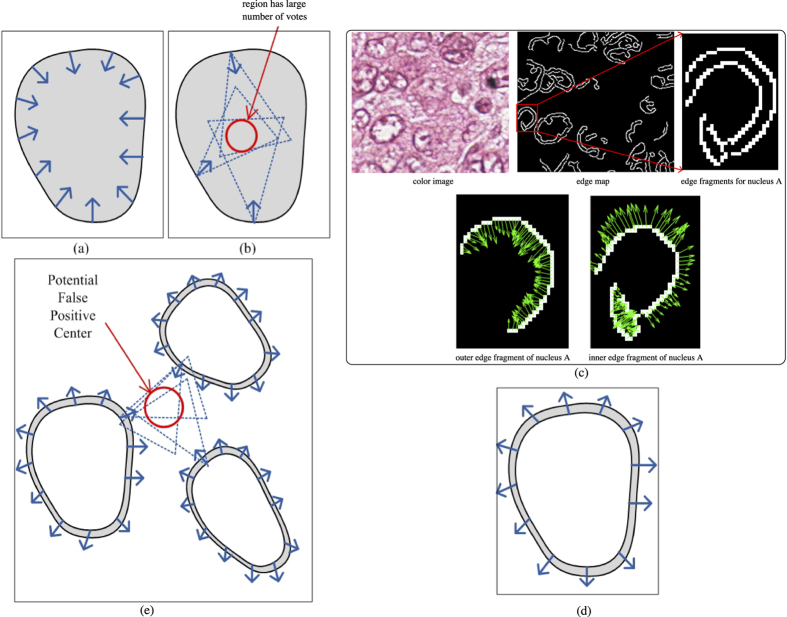
Illustrative examples for voting-based methods. (**a**) A synthetic nucleus with a few inverse gradient directions. (**b**) Illustration of region with a large number of votes. (**c**) An example of poorly stained tissue image with its edge map and edge fragments for one of the nuclei with the inverse gradients is shown. (**d**) An illustrative example of a nucleus with a hollow interior. (**e**) Examples of clustered nuclei. The thick blue arrows reflect the gradient information on the inner edge of the nuclei. Note that the outwards gradients are easily lead to potential false positive detections with voting based approaches.

The key idea behind voting based techniques is to cluster circular symmetries along the radial line/inverse gradient direction on an object’s contour in order to infer the center of the object of interest. An illustrative example is shown in Fig. 2(a,b). Figure 2(a) shows a synthetic phantom nucleus with foreground color as grey, and the background color in white. A few sample pixels/points on the nuclei contour with their inverse gradient directions are shown as blue arrows in Fig. 2. Figure 2(b) illustrates the voting procedure with three selected pixels on the contour. Note that for each pixel, a dotted triangle is used to represent an active voting area. The region where three voting areas converge can be thought of as a region with a high likelihood of containing a nuclear center.

Several effective symmetric voting-based techniques have been developed employing variants of the same principal. Parvin *et al*.^27^ proposed a multi-pass voting (MPV) method to calculate the centroid of overlapping nuclei. Qi *et al*.^22^ proposed a single pass voting (SPV) technique followed by a mean-shift procedure to calculate the seed points of overlapping nuclei. In order to further improve the efficiency of the approach, Xu *et al*.^24^ proposed a technique based on an elliptic descriptor and improved single pass voting for nuclei via a seed point detection scheme. This initial nuclear detection step was followed by a marker-controlled watershed algorithm to segment nuclei in H&E stained histology images. In practice, the MPV procedure tends to yield more accurate results compared to the SPV procedure in terms of nuclei detection. The SPV procedure may help improve overall efficiency of nuclear detection^24^, however, it needs an additional mean-shift clustering step to identify the local maxima in the voting map. This additional clustering step requires estimating additional parameters and increases overall model complexity.

Since existing voting-based techniques typically utilize edge features, nuclei with hollow interiors could result in incorrect voting and hence in generation of a spurious detection result. One example is shown in Fig. 2(c), where we can see a color image, its corresponding edge map and one of the nuclei, denoted as A. Nucleus A has a hollow interior so that it has two contours, an inner and an outer contour, which results in two edge fragments in the edge map (see second row of Fig. 2(c)). For the outer nuclear contour, the inverse gradients are pointing inwards, whereas for the inner nuclear contour, the inverse gradients are pointing outwards. As one may expect, the inverse gradient obtained from the inner contour minimally contributes towards identifying the nuclear centroid (because the active voting area appears to be outside the nucleus, while the nuclear center should be within the nucleus). Another synthetic example of a nucleus with a hollow interior is shown in Fig. 2(c), and a few inverse gradient directions are drawn on the inner contour. In most cases, those inverse gradients from the inner contour will lead to a spurious result in regions of clustered nuclei. In Fig. 2(e), three synthetic nuclei with hollow regions are shown. It is clear that due to the vicinity of these three nuclei, the highlighted red circle region has received a large number of votes and thus could lead to a potential false positive detection. In section, we will show that in real histopathologic images, existing voting-based techniques tend to generate many false positive detection results.

**Figure 3:**
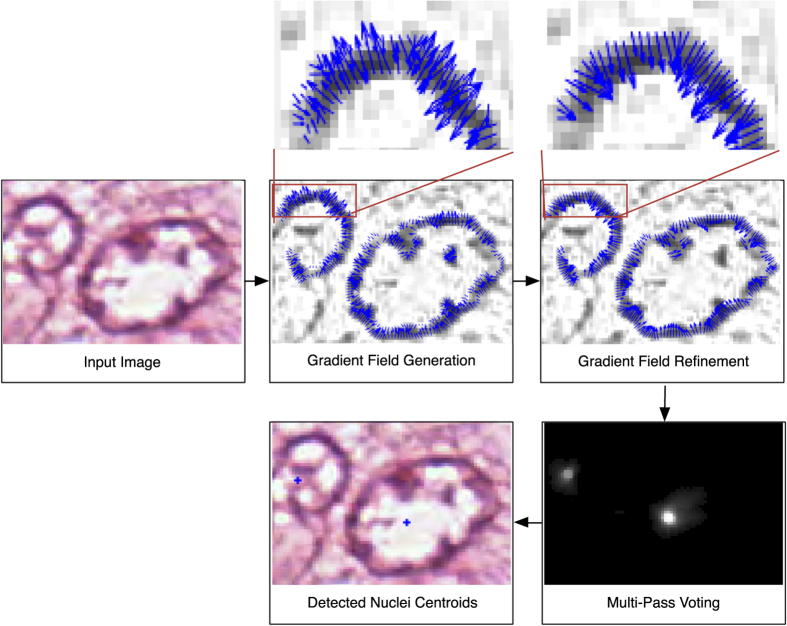
Schematic of MPAV. Given a color image, the gradient filed generation module generates a gradient field by using of image smoothing and edge detection. In the second module, the gradient field is refined, gradients whose direction are pointing outwards the outerior region of nuclei are removed or corrected. The refined gradient field is then utilized in the multi-pass voting module to guide each edge pixels for generating the nuclear voting map. Finally, a global threshold is applied on the voting map to obtain candidate nuclear centroids.

In this paper, we present a Multi-Pass Adaptive Voting (MPAV) method. The MPAV is a voting based technique which adaptively selects and refines the gradient information from the image to infer the location of nuclear centroids. The schematic for the MPAV is illustrated in Fig. 3. The MPAV consists of three modules: gradient field generation, refinement of the gradient field, and multi-pass voting. Given a color image, a gradient field is generated by using image smoothing and edge detection. In the second module, the gradient field is refined, gradients whose direction leads away from the center of the nuclei are removed or corrected. The refined gradient field is then utilized in a multi-pass voting module to guide each edge pixels for generating the nuclear voting map. Finally, a global threshold is applied on the voting map to obtain candidate nuclear centroids. The details of each module are discussed in the next section and the notations and symbols used in this paper are summarized in Table 2.

## Multi-Pass Adaptive Voting

**Figure 4:**
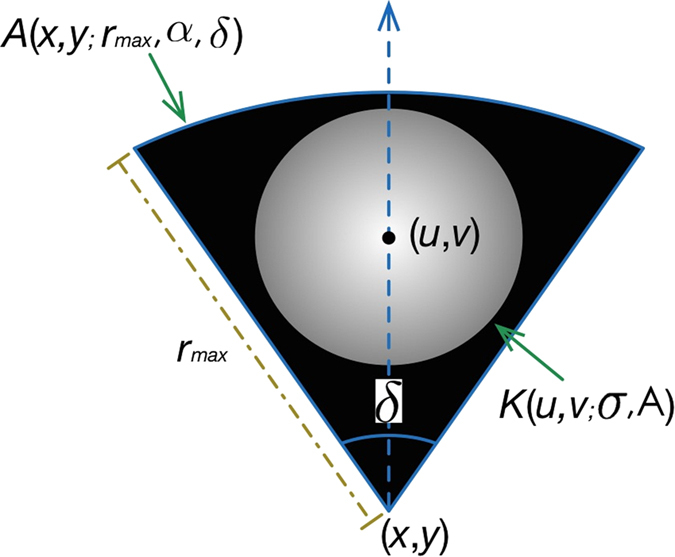
Illustration of an active voting area *A* with Gaussian kernel *K*.

For a pixel located at (*x*, *y*), an active voting area *A*(*x*, *y*; *r*_*min*_, *r*_*max*_, *α*, *δ*) is generated, where *r*_*min*_ and *r*_*max*_ indicate the radial range of the active voting area. *r*_*min*_ is usually set to 1 pixel without loss of reliability (an active voting area can now be represented as *A*(*x*, *y*; *r*_*max*_, *α*, *δ*)). Let *α* represents the inverse gradient direction at pixel (*x*, *y*); *δ* indicates the angular range. An active voting area is a fan-like area illustrated in Fig. 4. Within the voting area, a kernel, normally a 2-D Gaussian Kernel *K*(*u*, *v*; *σ*, *A*)^22^,^24^,^27^, is placed at (*u*, *v*) to diffuse the votes. That is, pixels close to (*u*, *v*) will have received a higher number of votes and vice versa.

### Gradient Field Generation

**Figure 5:**
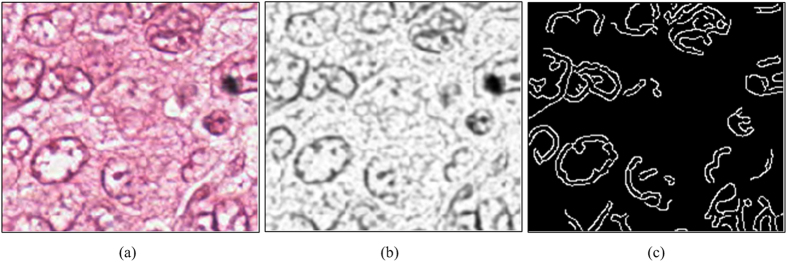
Creating the edge map. (**a**) Original color image; (**b**) smoothed red channel image; (**c**) edge map.

An unsupervised sparse non-negative matrix factorization (SNMF) based color deconvolution technique^38^ is first applied to separate different stains. We denote the single stain image as *I* and its 2-D image coordinates as (*x*, *y*). In order to reduce image noise, a smooth image *I*_*s*_ is obtained by convolving *I* with a Gaussian kernel 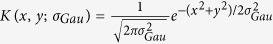, see Fig. 5(a,b) for examples.

The inverse gradient map 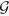 is generated using the following equations:


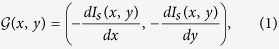


where 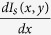 and 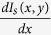 represent the gradients along the X and Y axes, respectively.

In this approach, an edge detection method based on Canny operator^39^ is applied to *I*_*s*_ to obtain nuclear edges. An example of edge detection is shown in Fig. 5(c), where white regions represent pixels that belong to nuclear edges. From here on, we only consider pixels that belong to nuclear edges. We denote the pixels set that belongs to a nuclear edge as *E*. An updated gradient map 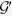 is then generated as follows:


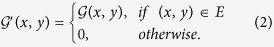


### Refinement of the Gradient Field

In the last step, we obtained an edge map that reflects which pixels will join in the voting procedure (shown in Fig. 5(c)). Since in the scenario that there are staining inconsistencies (see Fig. 2(c)), the inverse gradients on the inner edge fragment may not contribute to the voting of nuclear centroid and may introduce spurious votes in the voting map, we refine the inverse gradient of pixels that will join in the voting procedure. In other words, we do not blindly use all pixels and their corresponding gradient information during the voting step. Instead, we adaptively select the valid pixels and only use their associated gradient information during the voting stage. The steps corresponding to the gradient field refinement are described below.

#### Convex Hull Center Computation

**Figure 6:**
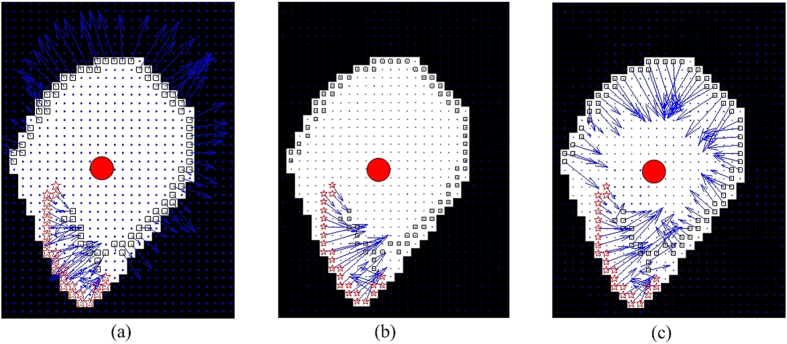
(**a**) Example of convex hull region for an edge fragment. The white region illustrates the convex hull. The solid red dot indicates the centroid of the convex hull. Small squares and stars represent the edge pixels, whereas the blues arrows represent the gradient information. Note that most of the inverse gradients are pointing out from the nuclear center. The edge pixels with invalid gradients are marked via squares, whereas the edge pixels with valid gradients are marked via red stars. (**b**) Illustration of the gradient refinement result using the first strategy, in which the gradient value of invalid gradient pixels are set to 0. (**c**) Illustration of gradient refinement result using the second strategy, in which the gradient sign of invalid gradient pixels are reversed.

For an edge fragment, *E*_*i*,*i*∈{1,…,*Q*}_, where *Q* is the total number of edge fragments in the edge map, we compute the corresponding convex hull, denoted as *H*_*i*_, and identify the center of the convex hull, denoted as *C*_*i*_. The convex hull region corresponding to the edge fragment in Fig. 2(c) is shown in Fig. 6(a). The red circle reflects the center of *C*_*i*_. The white region represents the convex hull region for the outer edge shown in Fig. 2(c), whereas the original pixels belonging to the edge fragment, i.e., pixels *p*(*x*, *y*) ∈ *E*_*i*_, are marked with rectangles and stars.

#### Angle Difference Computation

For a pixel in the current edge fragment, *p*(*x*, *y*) ∈ *E*_*i*_, we compute a vector, 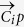, formed by the current pixel, *p*, and the center of *C*_*i*_. We then compute the angle difference *θ*(*x*, *y*) between the vector 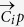 and inverse gradient direction as follows:


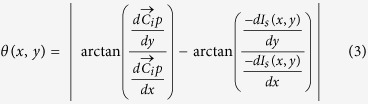


**Figure 7:**
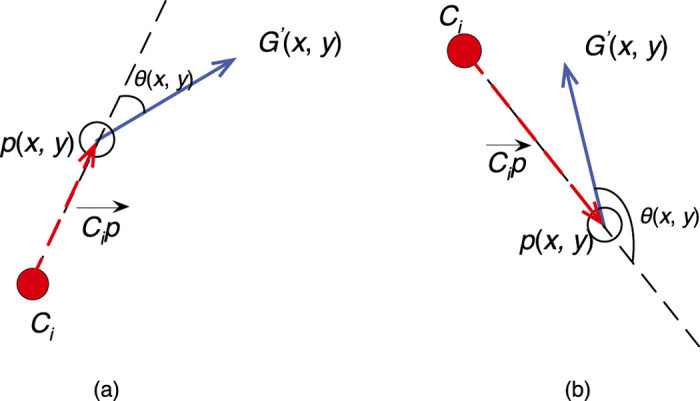
Illustrative example of angle difference computation. (**a**) A case where 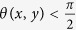. (**b**) A case where 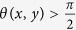.

Two illustrative examples of the procedure for angle difference computation are shown in Fig. 7(a,b), respectively, where a hollow circle represents a pixel *p*(*x*, *y*) ∈ *E*_*i*_ and a solid red circle represents the center of *C*_*i*_. The angle difference *θ*(*x*, *y*) between the vector 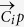 and inverse gradient *D*(*x*, *y*) (shown via the solid blue arrow) is also shown in Fig. 7(a,b).

#### Identifying Valid Voting Pixels

We employ the following heuristic to determine whether the gradient of a pixel is appropriate for participating in the voting strategy,


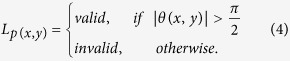


where *L*_*p*(*x*,*y*)_ denotes the label for pixel *p*(*x*, *y*). The rationale behind Eq. 4 is that if the inverse gradient points towards the hull centroid, it has a positive contribution towards the symmetry voting. On the other hand, if the inverse gradient points outwards from the hull centroid, it contributes negatively and may result in a false positive detection result. Illustrative examples are shown in Fig. 7. Figure 7(a) shows an example where the pixel is determined to be an invalid pixel, while Fig. 7(b) shows an example where the pixel is determined to be a valid pixel. In Fig. 6(a), all the invalid pixels are marked with black rectangles whereas all valid pixels are marked with red stars.

#### Invalid Voting Gradients Refinement

We present two gradients refinement strategies, Strategy 1 (GS1) and Strategy 2 (GS2), to refine the inverse gradient filed (we denote the MPAV with GS1 as 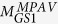, the MPAV with GS2 as 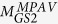, and the original MPV method as *M*^*MPV*^). GS1 simply ignores the pixels which are labeled as ‘invalid’. Mathematically, the refined inverse gradient map 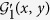 is then calculated as follows:


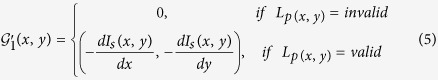


GS2 involves setting the inverse gradient directions of ‘invalid’ pixels to the reverse direction. Mathematically, the refined inverse gradient map 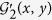 is calculated as follows:


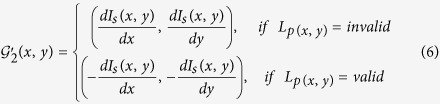


Figure 6(b,c) show the refinement result calculated by using Eqs 5 and 6, respectively. It is clear that for GS1 shown in Eq. 5, the inverse gradients of all invalid pixels are set to 0. For the GS2 shown in Eq. 6, the the directions of inverse gradients for all invalid pixels are reversed. GS1 ignores pixels with ‘invalid’ gradients and reduces the number of pixels that used in the voting procedure, whereas GS2 tries to refine the gradient direction of pixels with ‘invalid’ gradient and attempts to infer a more accurate result. We do not suggest that either of GS1 or GS2 is necessarily better than the another, rather they represent two alternative strategies which the user might want to invoke for a specific application.

### Multi-Pass Voting

The steps of MPV are summarized as follows.

#### Initialize voting parameters

Radial maximum range *r*_*max*_, maximum angular range *δ*_*max*_, and iteration number *N* are the voting parameters that need to be initialized. The angular ranges set is computed as follows,


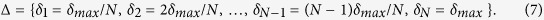


The initial voting directions *α* for all pixels are based on the inverse gradient map *G*′ (note that we have two different refinement strategies which correspond to two different refined inverse gradient maps 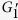 and 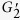, respectively). That is,


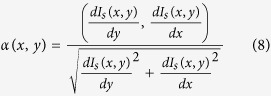


where 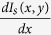 and 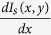 represent the gradients along the X and Y axes, respectively.

#### Update the vote image

For each pixel (*x*, *y*) ∈ *E* and *G*′(*x*, *y*) ≠ 0, compute the vote image *V*_*i*_, for the *i*th iteration, using Eq. 8. In Eq. 8, *K*(*u*, *v*; *σ*, *A*(*x*, *y*; *r*_*max*_, *α*_*i*_, *δ*_*i*_)) represents the kernel placed at location (*u*, *v*) within an active voting region *A*(*x*, *y*; *r*_*max*_, *α*_*i*_, *δ*_*i*_). Gradient map *G*′ can then be refined gradient map 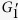 or 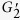.

#### Update voting direction

For each pixel (*x*, *y*) ∈ *E* and *G*′(*x*, *y*) ≠ 0, we employ the following equation to update the voting direction,


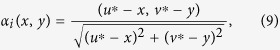


where


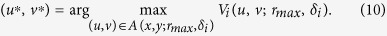


Intuitively, Eq. 9 involves updating the voting direction for pixel (*x*, *y*) with the pixel located at (u^*^, v^*^), which is the pixel that has the maximum vote value within the active voting region *A*, calculated in Eq. 10. In this way, we can perform the next pass/iteration of voting with a more precise voting direction, since the voting from the first iteration may be affected by the presence of noisy edge fragments that do not belong to the nuclei.

#### Iteration parameter adjustment

Using a smaller angular value, i.e., *δ*_*i*+1_, set *i* = *i* + 1, and repeat steps 2.3.2 to 2.3.4, until *i* = *N*. The reason to reduce the voting area angle is to reduce the size of voting area. The idea is that by doing this we can identify a more precise nuclear centroid candidate region.

**Figure 8:**
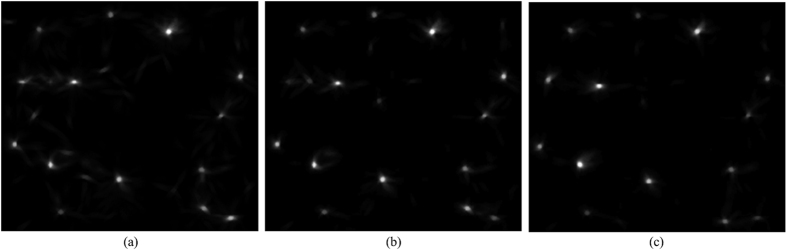
Voting images of the (**a**) *M*^*MPV*^; (**b**) 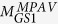; (**c**) 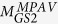.

**Figure 9:**
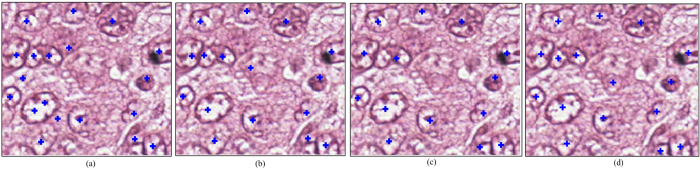
(**a**) Nuclear centers detected in *M*^*MPV*^; (**b**) 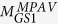; and (**c**) 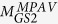; (**d**) manually labeled nuclei centers.

After *N* iterations, a final vote image *V*_*N*_ is obtained. The final vote image *V*_*N*_ contains the voting information from all pixels that join in the voting procedure. Figure 8 shows the voting image for the *M*^*MPV*^, and 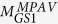 and 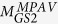, respectively. The bright regions in Fig. 8 correspond to a high vote values and therefore indicate potential nuclear centers. On the other hand, black regions indicate fewer votes and background location. It is clear that all voting images for each of *M*^*MPV*^, 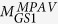 and 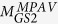 have high values near the ground truth nuclear centers (the manually labeled nuclei centers are shown in Fig. 9(d)). However, it should be noted that in the voting image obtained by *M*^*MPV*^, there are many regions with a high vote value. Compared to the vote image obtained by *M*^*MPV*^, 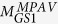 and 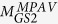 appear to yield more specific and accurate detection results.

Finally, a global threshold *T*_*D*_ is applied to the voting image *V*_*N*_ to obtain nuclear centers. Specifically, we use the automated Ostu’s threshold method^40^ to determine a global threshold *T*_*D*_. We then apply this threshold on *V*_*N*_ to obtain a binary image. The centroids of the connected components are then identified as the final nuclear centers. Figure 9(a–c) show the final nuclear centers, highlighted with blue crosses, obtained from each of *M*^*MPV*^, 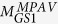, and 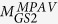, respectively. The manually labeled nuclear centers are shown in Fig. 9(d).

## Experimental Setup

### Image Dataset

**Figure 10:**
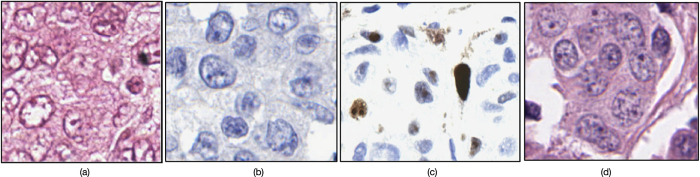
Sample images from four datasets. (**a**) A (Hematoxylin&Eosin), (**b**) B (CD31&Hematoxylin), (**c**) C (Ki-67), (**d**) D(Hematoxylin&Eosin).

We evaluated the 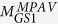 and 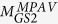 on three different histopathologic image datasets. Table 3 describes the properties of these datasets. Dataset A is a publicly available histopathologic image dataset (H&E-stained digital images of breast cancer tissue slides)^41^. Dataset B consists of 10 breast cancer images stained with CD31 antibody and hematoxylin^13^. Dataset C consists of 5 skin cancer images stained with Ki-67^42^. Dataset D consists of 21 breast cancer tissue images stained with H&E. Note that the dataset A and D contain images with noisy background and nuclei with heterogeneous appearance due to the uneven staining. The nuclei locations were manually labeled by two experts, one a breast cancer pathologist with over 10 years of experience and the second an image computing scientist with over 3 years of experience in working with breast pathology. Figure 10 illustrates example images from A, B, C, and D, respectively.

### Evaluation Metrics

The main objective of the evaluation was to determine if the nuclear centers detected by the MPAV technique are concordant with the manually labeled nuclear centers. We calculate the center of each segmented region obtained by the automated technique. A nucleus was identified as having been correctly detected if its center is within a range of 15 pixels, i.e., about 3.6825 *μ*m, of the manually labeled nuclei center location.

Denoting 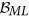 as the total number of manually labeled nuclei centers, 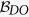 as the total number of detected nuclei centers, 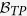 as the number of true-positives, (i.e., correctly detected objects compared to the manually labeled ones), 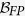 as the number of false-positives. (i.e., falsely detected objects compared to the manually labeled ones).

The performance is evaluated with respect to recall (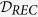), precision (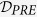), and F-measure (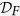) which are defined as follows:


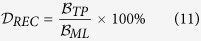



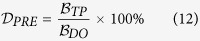



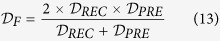


In order to evaluate detection performance, all the nuclei detection techniques (see comparative methods below in Section IV-C) were evaluated via the precision-versus-recall rate curve (PRC). In a PRC, the horizontal and vertical axes represents 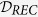 and 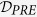, respectively. The closer the PR curve to the upper left corner, the better the corresponding detection method. Area under the PRC curve (AUC) is also calculated. The minimum and maximum value of a AUC is 0 and 1, corresponding in turn to the worst and best possible detection results. Note that the PRC for each of *M*^*SPV*^, 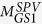, and 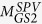, *M*^*MPV*^, 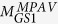, and 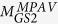 is calculated by tuning a global threshold *T*_*D*_ on all *V*_*N*_ for all the images in dataset. Assuming the value range in *V*_*N*_ is [0, 1], we use a set of value varying from 0 to 1 with a resolution of 0.05 for the *T*_*D*_. The detection performance of applied a *T*_*D*_ corresponds to a point on the PRC curve.

### Experimental Design and Comparative Strategy

#### Performance comparison with existing voting based techniques

We evaluated the performance of the original SPV technique^24^ (denoted as *M*^*SPV*^) and its enhanced version by adaptively choosing and refining the gradient information using 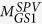, and 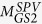, respectively. We also evaluated the performance of *M*^*MPV*^ and the enhanced versions 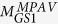, and 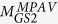, respectively. For all the voting based methods, we set *r*_*min*_ = 1, *r*_*max*_ = 40, *σ* = 4; for SPV methods, 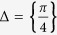; for *M*^*MPV*^, 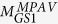, and 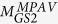, we set the voting iteration number *N* to 3, 
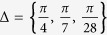
 (More details on parameters selection in initial processing and the parameters selection of voting area please refer to the supplementary material A and B).

#### Performance comparison with DL based technique

We also compared the detection performance of 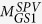, and 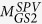 with a DL based technique, SSAE (denoted as *M*^*SSAE*^), developed by Xu *et al*.^13^. The *M*^*SSAE*^ is a 4 layers deep neural network. The input patch size used in *M*^*SSAE*^ was 34 × 34 pixels, therefore, the input layer receives 34^2^ × 3 input units. The first and second hidden layers have 400 and 255 hidden units, respectively. The output layer is a soft-max function which classifies the input patches as nucleus or non-nucleus. The *M*^*SSAE*^ was trained with a different set, other than datasets A, B, and C, of 37 H&E images of 2200 × 2200 pixels at 40x magnification^13^.

## Results and Discussions

### Performance comparison with existing voting based techniques

#### Qualitative results

**Figure 11:**
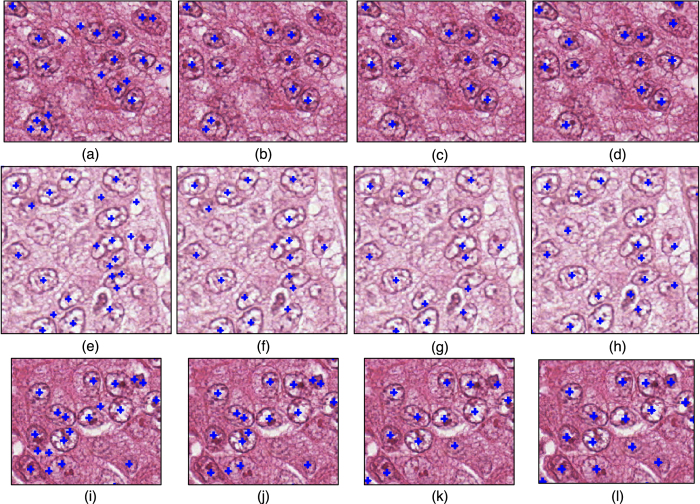
Three examples, from Dataset A, for the comparison of nuclear seed point detection via *M*^*MPV*^, 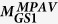, and 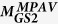. (**a,e,i**) *M*^*MPV*^, (**b,f,j**) 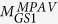. (**c,g,k**) 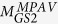. (**d,h,l**) show the manually labeled nuclei seed points.

Figure 11 presents three examples illustrating *M*^*MPV*^, 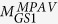, and 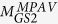. The manually labeled nuclei centers are shown in the last column, whereas automatically calculated nuclei centers are shown in the first three columns (all nuclei centers are marked with blue crosses) for *M*^*MPV*^, 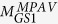, and 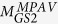. It is clear that *M*^*MPV*^ tends to generate false positives errors in regions where nuclei are clustered and image noise is present. This is because neighboring pixels from different nuclear edge fragments generate spurious regions with high vote value. 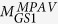 and 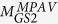 are able to generate more accurate results with fewer false positives (automatically generated nuclear centers which are not concordant with the manually labeled nuclear centers).

#### Quantitative results

**Figure 12:**
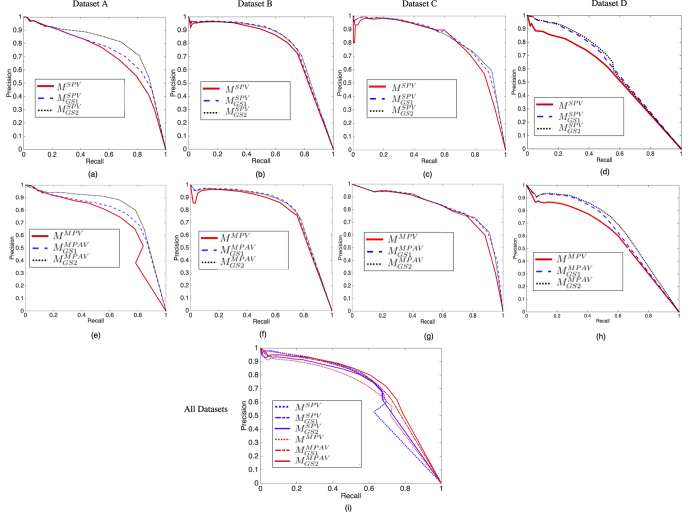
The performance comparison of SPV and MPAV in terms of PRC, the x-axis represents the 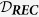 whereas the y-axis represents the 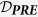. The first row shows the performance of *M*^*SPV*^^24^ compared to 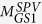 and 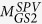 (**a–d**). The second row shows the performance of *M*^*MPV*^ compared to 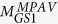 and 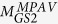 (**e–h**). (**g**) shows the overall performance, for datasets A, B, C, and D, comparison of *M*^*MPV*^^27^, *M*^*SPV*^^24^, 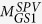, 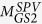, 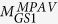, and 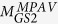. Note that only the voting based methods are illustrated in this figure.

The detection performance in terms of PRC for datasets A, B, C, and D are shown in Fig. 12. The first row shows the performance of *M*^*SPV*^^24^ compared to the 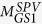 and 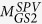 techniques (Fig. 12(a–c)). The second row shows the performance of *M*^*MPV*^^27^ compared to 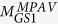 and 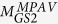 (Fig. 12(d–f)). Figure 12(g) shows the overall detection performance comparison for methods *M*^*MPV*^, *M*^*SPV*^, 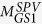, 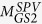, 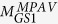, and 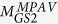 on datasets A, B, C and D.

The nuclei detection results for dataset A and D are shown in Fig. 12(a,e) and (d,h), respectively. 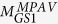 and 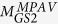 achieve better performance in such noisy images since they adaptively refine the gradient information for voting. In Fig. 12(b,f), it may be observed that the improvements obtained by the gradient refinement strategies for dataset B are less substantial. In Fig. 12(c,g), the detection performances are almost identical. This is because the images in datasets B and C contain less noise that may affect the voting procedure. The refinement procedure thus has little effect on the voting result. In dataset A and D, there are many clustered nuclei, and hence, 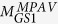 and 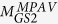 yield substantial gains in performance over *M*^*MPV*^, *M*^*SPV*^, 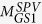, and 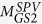. Figure 12(g) shows the detection performance comparison of *M*^*MPV*^^27^, *M*^*SPV*^^24^, 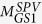, 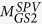, and the 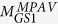 and 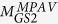 for datasets A, B, and C, comprising a total of 11844 annotated nuclei. It is worth nothing that for 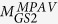, where the gradients direction are reversed (in Eq. 6), provides the best performance.

In Table 4, we present the quantitative result of *M*^*MPV*^, *M*^*SPV*^, 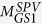, 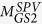, 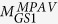, and 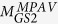 in terms of the best 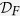 and its associated 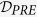 and 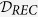 (fifth, thrid and fourth column, respectively). The area under the PRC curve for all plots, shown in Fig. 12, are also calculated and presented in Table 4.

### **Performance comparison with DL based technique** ***M*** ^***SSAE***^

#### Qualitative results

**Figure 13:**
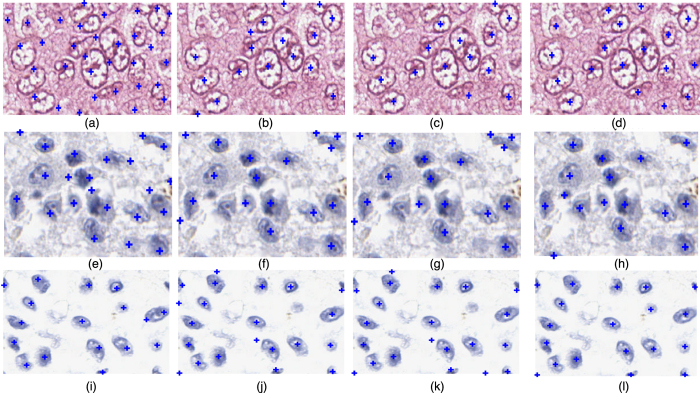
Three visual examples for the comparison (the nuclei seed points are indicated with blue crosses). Each row shows one example for datasets A, B, and C, respectively. The first to the fourth columns show the detection result obtained by *M*^*SSAE*^, 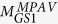, 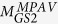, and manually labeled exemplars.

Figure 13 presents three visual examples for the performance comparison of methods *M*^*SSAE*^, 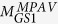, and 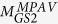 on datasets A, B, and C. The manually labeled nuclei centers are shown in the last column, whereas automatically calculated nuclei centers for methods *M*^*SSAE*^, 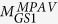, and 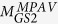 are shown in the first three columns (all nuclear centers are marked with blue crosses). An example image from dataset A is shown in the first row, *M*^*SSAE*^ appears to produce a number of false positive detection results for this poorly stained image. Additionally *M*^*SSAE*^ is prone to treat the boundary point as the nuclei center due to the heterogeneous intensity distribution within the nuclei and nuclei boundaries. In the second row, one may observe that the MPAV appears to produce more false positive errors for nuclei that have a thin and long shape (see Fig. 13(f,g)).

#### Quantitative results

**Figure 14:**
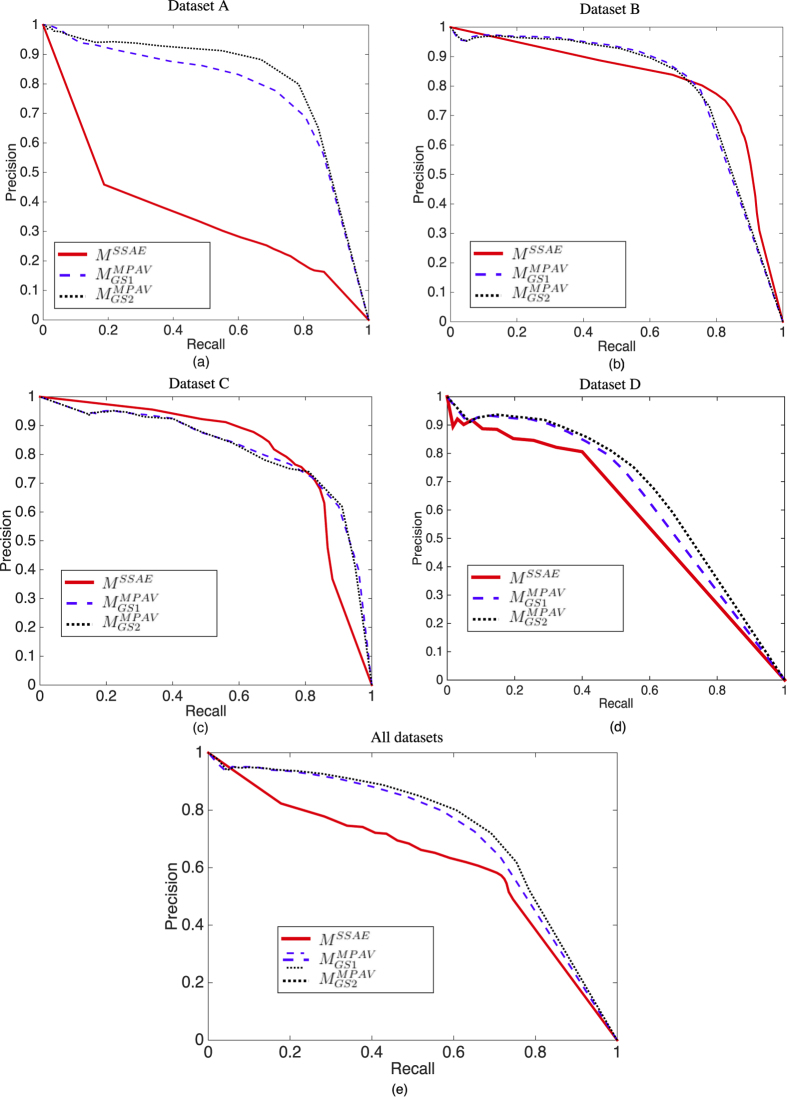
The performance comparison of *M*^*SSAE*^ with 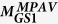 and 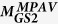. (**a–c**) show the PRC curves of *M*^*SSAE*^, 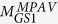, and 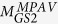 for datasets A, B, and C, respectively. (**d**) shows the PRC curves of *M*^*SSAE*^, 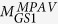, and 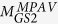 for datasets A, B, C, and D.

The detection performance in terms of PRC for all 4 datasets are shown in Fig. 14. As shown in Fig. 14(a), the nuclei detection result for *M*^*SSAE*^ on dataset A is poor, since the *M*^*SSAE*^ is learned from dataset with well stained nuclei (and we also trained *M*^*SSAE*^ with dataset A and then tested on dataset A, the result is even worse, possibly on account of too few training instances). In Fig. 14(c), *M*^*SSAE*^ marginally outperforms 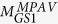 and 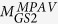. However, 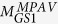 and 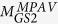, provide consistently good results on all four datasets. 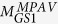 and 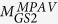 may generate spurious result if the nuclear size is relatively large, since the voting is based on a predefined range of active voting area, i.e., *r*_*max*_ and *r*_*min*_. However, the number of abnormally large nuclei is small in most of the images, most of the nuclei generally fall within a fixed size range. 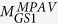 and 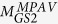 are unsupervised methods that do not require training, whereas the *M*^*SSAE*^ requires a large number of samples to learn the underlying patterns, and the heterogeneous appearance of nuclei in different stain conditions may hence affect the learned features and final detection performance.

Table 5 presents corresponding quantitative result of *M*^*SSAE*^, 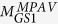, and 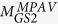 in terms of the best 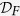 and its associated 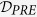 and 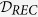 for datasets A, B, C, and D. The area under the PRC curve, for the curves shown in Fig. 14 are presented in the last column of Table 5.

### Statistics evaluation of detection results

**Figure 15:**
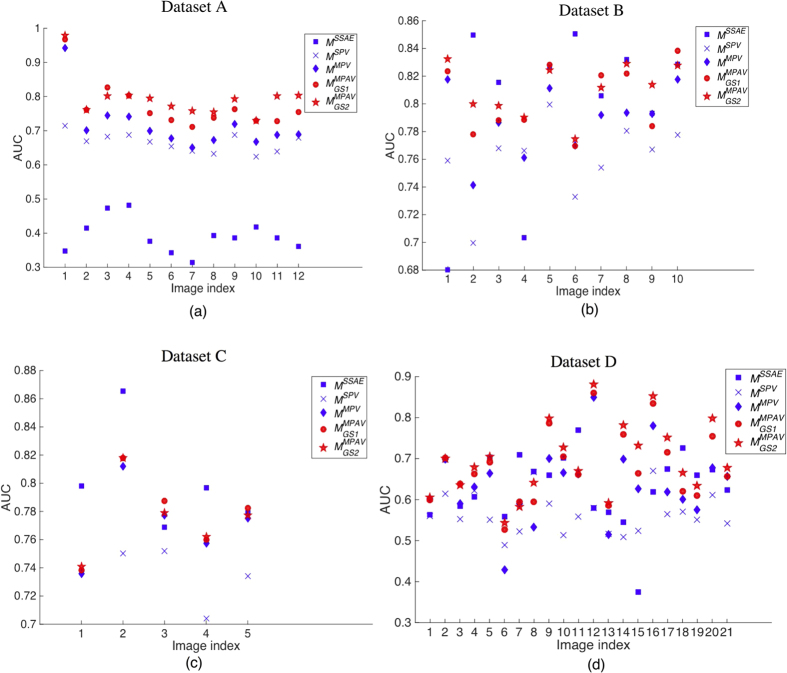
(**a–d**) Show the cloud plots of AUC for every image in dataset A–D respectively. Each point in the plot corresponds to a detection performance in terms of AUC of an image for a certain technique. The paired t-test p-values between each technique are shown in Table 6. It is observed that in dataset A and D, which contains noisy images, the MPAV technique is significant better than *M*^*SSAE*^, *M*^*SPV*^, and *M*^*MPV*^ techniques. In dataset B and C, there is no significant different between the *M*^*MPAV*^ and the *M*^*SSAE*^.

We calculated the AUC for every image in the datasets and plotted the AUCs as a point for each image, as shown in Fig. 15. Figure 15(a–d) show the AUC cloud plots for dataset A–D respectively. A higher AUC corresponds to a better performance. From these cloud plots, one may observe the detection performance for each image within a dataset. The two-sample t-test p-values between each technique are calculated based on the AUCs obtain from different techniques and presented in Table 6. In this work, we used 0.05 as the significance level for the p-valudes to determine statistical significance. One may observe that in dataset A and D, the 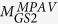 is significantly different than other techniques. Related to Figs 12 and 14, the 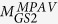 is actually provide “significantly” better results than *M*^*SSAE*^, *M*^*SPV*^, and *M*^*MPV*^ techniques in the noisy images. As in the cases of dataset B and C, the 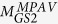 technique is not significantly different than the *M*^*SSAE*^ techniques. Note that in Figs 12 and 14, we calculated the PRC for all the images within each image dataset, whereas in Fig. 15, we generated the PRC and calculated the AUC for each image whithin the dataset spearately.

## Concluding Remarks

In this paper, a Multi-Pass Adaptive Voting technique was presented for automated nuclei detection on histopathological images. While most previous nuclear detection approaches tend to work well on well stained images, these approaches tend to fail on unevenly and imprecisely stained images. In practice, due to the variations of staining and slide preparation methods, a number of images tend to stain poorly. The MPAV is able to adaptively ignore or refine and identify the pixels that may lead to inaccurate nuclei centers in the voting procedure. To show the effectiveness of the MPAV, we compared it with existing voting based methods as well as a state-of-the-art deep learning method. The main contribution of the presented MPAV is to adaptively utilize the gradient information of pixels on edge fragments to generate more accurate detection results by exploiting the symmetry of nuclei. Both qualitative and quantitative evaluation results show that the MPAV appears to be able to address many of the limitations of existing voting based techniques for nuclei detection in unevenly and imprecisely stained histology images. While compared to the DL-based method, MPAV provides a consistently superior detection performance whereas the DL method requires more training samples for poorly stained images. Note that the MPAV approach aims solely to detect nuclei and not to explicitly segment them, though the result of detection could serve as the initialization for other (e.g., watershed^24^) nuclear segmentation approaches. However, our approach does have its limitations. Firstly, due to the noise and extraneous tissue components in the image, there may exist some noisy edge pixels outside the true nuclei on the edge segments. In these cases, the estimated center of the convex hull may fall outside the true nuclei, which leads to an inaccurate determination of which pixels are either valid or invalid for the purposes of voting. In our experiments, the number of such cases was small. Secondly, while our new gradient refinement strategy is able to improve the detection performance in noisy histopathological images, for relatively good quality images with well stained nuclei, like datasets B and C shown in Fig. 10(b,c), there is marginal or no improvement (detection result shown in Fig. 12(b,e,c,f)). However, the MPAV technique only has a marginal increment in terms of computational cost compared to the original MPV method (A Matlab implementation of MPAV algorithm is provided in the supplementary material C). In future work, we aim to integrate the MPAV with segmentation methods such as level sets and active contour schemes to explicitly extract nuclear boundaries.

## Additional Information

**How to cite this article**: Lu, C. *et al*. Multi-Pass Adaptive Voting for Nuclei Detection in Histopathlogical Images. *Sci. Rep*. **6**, 33985; doi: 10.1038/srep33985 (2016).

## Supplementary Material

Supplementary Information

## Figures and Tables

**Figure 1:**
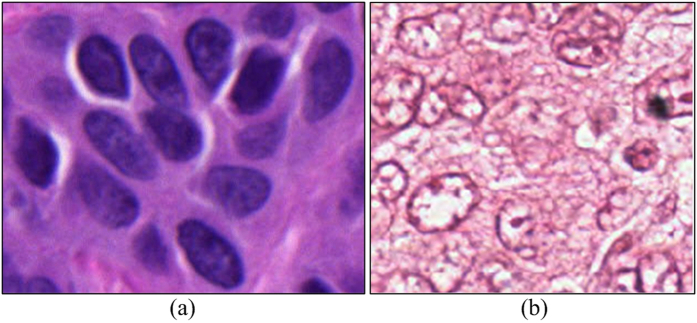
Examples of Hematoxylin and Eosin (H&E) stained histopathological images. (**a**) A sample image with nuclei that are well stained and have clear boundaries. (**b**) A sample image with nuclei that have ill-defined nuclear contour due to uneven staining.

**Table 1:** Typical existing methods for nulcei detection.

Category	Nuclei detection methods
Color-based	blue ratio^28^
	color clustering^29^
	local adaptive thresholding^25^
	LoG filter^31^
Edge-based	adaptive H-minima transform^31^
	watershed^19^,^21^,^24^
	gradient & mophological operation^33^
	circular Hough transform^34^
Texture-based	diffused gradient vector field^35^
Deep learning-based	convolutional autoencoder neural network^36^
	stacked sparse autoencoder^13^
Voting-based	multiple passes voting^27^
	single pass voting^22^,^24^
	region-based voting^37^
	**multi-pass adaptive voting**

**Table 2:** Notation and symbols used in this paper.

Symbols	Description
*I*	image
(*x*, *y*)	pixel location/coordinate in image
*σ*_*Gau*_	the parameter of Gaussian filtering in initial processing
*T*_*Canny*_	the threshold of Canny edge detection in initial processing
*r*_*min*_, *r*_*max*_	radial range of active voting area
*Q*	total number of edge fragments in the edge map
*α*	inverse gradient direction
*δ*	angular range of active voting area
*A*(*x*, *y*; *r*_*min*_, *r*_*max*_, *α*, *δ*)	active voting area
(*u*, *v*)	center of 2-D Gaussian Kernel
*K*(*u*, *v*; *σ*, *A*)	2-D Gaussian Kernel
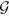	inverse gradient map
*E*	pixels set that belongs to nuclear edges
*H*_*i*_	convex hull for the *i*th edge fragment
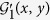	refined inverse gradient map with strategy 1 (GS1)
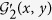	refined inverse gradient map with strategy 2 (GS2)
*N*	variable for iteration count for voting
*V*_*i*_	vote image at iteration *i*
*V*_*N*_	final vote image at iteration *N*
*T*_*D*_	global threshold applied on voting map/probablity map
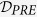	precision rate
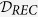	recall rate
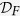	F-measure
*M*^*SPV*^	original single pass voting method^24^
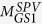	single pass voting method in conjuntion with GS1
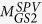	single pass voting method in conjuntion with GS2
*M*^*MPV*^	original multiple pass voting method^27^
*M*^*SSAE*^	stacked sparse autoencoder (SSAE) method^13^
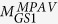	multi-pass adaptive voting method with GS1
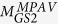	multi-pass adaptive voting method with GS2

**Table 3:** Image datasets used for evaluation.

Name	Site	Stain	Resolution(*μ*m/pixel)	No. of Images	No. of Nuclei
Dataset A	Breast	Hematoxylin&Eosin	0.2455	12	4598
Dataset B	Breast	CD31&Hematoxylin^13^	0.2514	10	5248
Dataset C	Skin	Ki-67^42^	0.2475	5	1998
Dataset D	Breast	Hematoxylin&Eosin	0.2455	21	5859

**Table 4:** Performance comparison of *M*^*SPV*^, 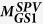, and 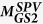, *M*^*MPV*^, 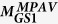, and 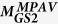, in terms of 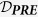, 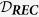, 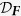, and AUC, evaluated evaluated on datasets A, B, and C.

Dataset	Techniques	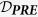 (%)	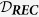 (%)	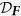 (%)	AUC
Dataset A	*M*^*SPV*^	67.59	67.44	67.51	0.72
	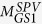	70.26	71.37	70.81	0.75
	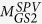	70.91	**81.33**	**75.76**	**0.79**
	*M*^*MPV*^	65.27	77.86	71.01	0.72
	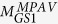	77.64	71.53	74.46	0.77
	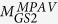	**79.94**	**78.46**	**79.19**	**0.82**
Dataset B	*M*^*SPV*^	**80.51**	**68.34**	**73.93**	**0.77**
	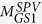	75.81	74.89	75.35	0.79
	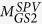	79.54	72.04	75.60	0.79
	*M*^*MPV*^	75.37	**75.36**	**75.36**	**0.78**
	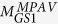	78.57	75.27	**76.88**	**0.80**
	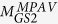	79.29	73.80	76.44	**0.80**
Dataset C	*M*^*SPV*^	72.00	76.00	73.95	0.74
	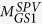	73.00	78.00	75.42	0.75
	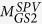	73.00	79.00	75.88	0.75
	*M*^*MPV*^	**76.43**	**74.99**	**75.70**	**0.77**
	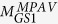	72.00	**82.00**	**76.68**	**0.77**
	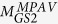	74.00	81.00	**77.34**	**0.77**
Dataset D	*M*^*SPV*^	60.58	52.70	56.37	0.56
	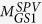	64.94	55.44	59.81	0.61
	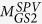	66.63	51.31	57.98	0.62
	*M*^*MPV*^	60.40	59.76	60.08	0.60
	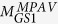	66.01	57.85	61.66	0.64
	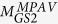	**67.65**	**61.15**	**64.24**	**0.67**
All	*M*^*SPV*^	64.33	66.93	65.60	0.66
	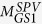	74.33	62.58	67.95	0.68
	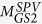	75.45	62.43	68.32	0.70
	*M*^*MPV*^	**75.62**	**61.53**	**67.85**	**0.67**
	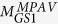	72.64	65.09	68.65	0.71
	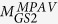	72.02	**69.16**	**70.56**	**0.73**

The highest performance for each dataset for each metric is shown in bold.

**Table 5:** Performance comparison of *M*^*SSAE*^, 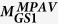, and 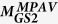, in terms of 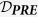, 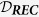, 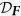, and AUC, evaluated on datasets A, B, and C.

Dataset	Techniques	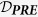 (%)	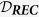 (%)	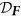 (%)	AUC
Dataset A	*M*^*SSAE*^	33.60	47.53	39.37	0.36
	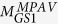	77.64	71.53	74.46	0.77
	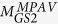	**79.94**	**78.46**	**79.19**	**0.82**
Dataset B	*M*^*SSAE*^	75.07	**82.64**	**78.67**	**0.81**
	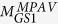	78.57	75.27	76.88	0.80
	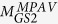	**79.29**	**73.80**	**76.44**	**0.80**
Dataset C	*M*^*SSAE*^	**75.63**	**78.75**	**77.16**	**0.81**
	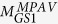	72.00	**82.00**	**76.68**	**0.77**
	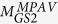	74.00	81.00	**77.34**	**0.77**
Dataset D	*M*^*SSAE*^	**80.58**	**39.99**	**53.45**	**0.57**
	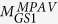	66.01	57.85	61.66	0.64
	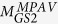	67.65	**61.15**	**64.24**	**0.67**
All	*M*^*SSAE*^	58.21	**70.60**	**63.81**	**0.62**
	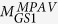	72.64	65.09	68.65	0.71
	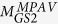	**72.02**	**69.16**	**70.56**	**0.73**

The highest performance for each dataset for each metric is shown in bold.

**Table 6:** Statistics evaluation of *M*^*SSAE*^, *M*^*SPV*^, *M*^*MPV*^, 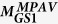, and 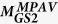, in terms of AUC, evaluated on datasets A, B, C, and D.

Dataset	Techniques	*M*^*SSAE*^	*M*^*SPV*^	*M*^*MPV*^	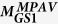	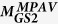
Dataset A	*M*^*SSAE*^	1	**5.83 × 10^−14^**	**2.50 × 10^−11^**	**2.77 × 10^−13^**	**2.15 × 10^−14^**
	*M*^*SPV*^	**5.83 × 10^−14^**	1	**4.00 × 10^−2^**	**5.51 × 10^−5^**	**1.14 × 10^−6^**
	*M*^*MPV*^	**2.50 × 10^−11^**	**4.00 × 10^−2^**	1	7.00 × 10^−2^	**1.00 × 10^−2^**
	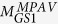	**2.77 × 10^−13^**	**5.51 × 10^−5^**	7.00 × 10^−2^	1	3.90 × 10^−1^
	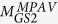	**2.15 × 10^−14^**	**1.14 × 10^−6^**	**1.00 × 10^−2^**	3.90 × 10^−1^	1
Dataset B	*M*^*SSAE*^	1	8.00 × 10^−2^	6.20 × 10^−1^	7.90 × 10^−1^	5.60 × 10^−1^
	*M*^*SPV*^	8.00 × 10^−2^	1	**2.88 × 10^−2^**	**1.60 × 10^−3^**	**1.81 × 10^−4^**
	*M*^*MPV*^	6.20 × 10^−1^	**2.88 × 10^−2^**	1	1.80 × 10^−1^	**4.08 × 10^−2^**
	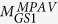	7.90 × 10^−1^	**1.60 × 10^−3^**	1.80 × 10^−1^	1	5.40 × 10^−1^
	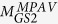	5.60 × 10^−1^	**1.81 × 10^−4^**	**4.08 × 10^−2^**	5.40 × 10^−1^	1
Dataset C	*M*^*SSAE*^	1	**8.40 × 10^−3^**	1.90 × 10^−1^	2.90 × 10^−1^	2.50 × 10^−1^
	*M*^*SPV*^	**8.40 × 10^−3^**	1	**4.81 × 10^−2^**	**3.20 × 10^−2^**	
	*M*^*MPV*^	1.90 × 10^−1^	**4.81 × 10^−2^**	1	7.70 × 10^−2^	8.30 × 10^−1^
	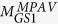	2.90 × 10^−1^	**3.20 × 10^−2^**	7.70 × 10^−2^	1	9.30 × 10^−1^
	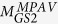	2.50 × 10^−1^	**3.20 × 10^−2^**	8.30 × 10^−1^	9.30 × 10^−1^	1
Dataset D	*M*^*SSAE*^	1	**4.20 × 10^−3^**	8.70 × 10^−1^	9.00 × 10^−2^	**2.00 × 10^−2^**
	*M*^*SPV*^	**4.20 × 10^−3^**	1	**3.00 × 10^−3^**	**6.81 × 10^−6^**	**5.96 × 10^−7^**
	*M*^*MPV*^	8.70 × 10^−1^	**3.00 × 10^−3^**	1	1.40 × 10^−1^	**3.00 × 10^−2^**
	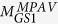	9.00 × 10^−2^	**6.81 × 10^−6^**	1.40 × 10^−1^	1	4.50 × 10^−1^
	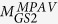	**2.00 × 10^−2^**	**5.96 × 10^−7^**	**3.00 × 10^−2^**	4.50 × 10^−1^	1

A two sample t-test is performed between each technique, the p-values are shown in the table. The p-values that are smaller than 0.05 are shown in bold, which is considered to be statistically significant in this work.
