# Supplementary material for: Multi-Pass Adaptive Voting for Nuclei Detection in Histopathological Images
Source: Sci Rep. 2016 Oct 3;6:33985. doi: 10.1038/srep33985 (PMC5046183; doi:10.1038/srep33985)
Supplement: Supplementary Information [file srep33985-s1.pdf]

Multi-Pass Adaptive Voting for Nuclei Detection in Histopathological Images

Supplementary information

Cheng Lu, Hongming Xu, Jun Xu, Hannah Gilmore, Mrinal Mandal, and Anant Madabhush

S1: Parameters Selection in Initial processing

In the initial processing, there are two parameters involved, one with the Gaussian filter (denoted as σ_{Gau}) and the other with the Canny filter for edge detection (denoted as T_{Canny}). σ_{Gau} is the width of the Gaussian filter, σ_{Gau} is generally an odd integer for image smoothing. The higher the value of σ_{Gau}, the smoother the output image. T_{Canny} is the edge threshold used for Canny filtering. In Canny filtering, the gradient of the image is first computed. Based on the gradient magnitude map, T_{Canny} maybe set to a high value for retaining all high gradient magnitude pixels, while T_{Canny} x 0.4 (lower threshold) may be used to remove all the low gradient magnitude pixels. A connectivity check is performed to see if the pixels with intermediate gradient magnitude are connected to the high gradient magnitude pixels. The resulting pixels form the subsequent edge map. The value for T_{Canny} is set relative to the highest value of the gradient magnitude within the image.

We performed an experiment on evaluating σ_{Gau} and T_{Canny} with respect to the AUC. In the experiment, $\sigma_{Gau} \in [3,5,7,9,11,13]$, $T_{Canny} \in [0.45, 0.55,0.65,0.75,0.85]$, and the AUC is evaluated within Dataset A. AUC is a function of different parameters (σ_{Gau} and T_{Canny}), shown in a 3D plot in Figure 1. One may observe that the performance is less affected by the Gaussian smoothing operation. The MPAV technique achieves max AUC when T_{Canny} =0.65. And the AUC maintains high when T_{Canny} is in the range of 0.55 to 0.75. The reason is that if T_{Canny} is set to a small value, a large number of noisy pixels that do not belong to the nuclei edges are conflated which adversely affects the voting inference. On the other hand, increasing the value of T_{Canny} will result in a decrease in the number of noisy pixels, potentially leading to better voting inferences being made. However, if T_{Canny} is too high, very few pixels will be engaged in the voting process, in turn leading to lower voting accuracy (as shown in Figure 2). A detailed explanation of this Figure and the accompanying explanation are included in the supplementary material section.

S2: Parameters Selection of Voting Area

The size of the voting area is mainly controlled by two parameters r_{max} and Δ. r_{max} determines the voting range, that is, larger the r_{max}, the larger the corresponding voting area. For the task of nuclei detection, r_{max} can be set to a value close to the diameter of a typical nucleus. For example, for a tissue slide digitized at 40x magnification, r_{max} can be set to 40 pixels. Δ is a set of angular values, e.g., we set $\Delta=(\frac{\pi}{4}, \frac{\pi}{7}, \frac{\pi}{28})$. Each angular value in Δ will determine the size of fan-like voting area at each iteration. Smaller the angular value, smaller the voting area. Since the voting method uses the convergence/overlapping of voting area to infer the centroids of nuclei, the initial angular value cannot be set to a large value. For example, if the number of iterations N=3, then we set $\Delta=(\frac{\pi}{4}, \frac{\pi}{7}, \frac{\pi}{28})$, note that the number of iterations N is associated with each value in Δ. The initial angle is set to $\frac{\pi}{4}$. As the number of iterations increase, the angular value decreases, and the size of the corresponding voting area decreases. This results in a narrower convergence region for voting with subsequent iterations. An illustration of V_N. with different voting angles and at different iterations is shown in Figure 3.

S3: Matlab implementation of the MPAV method.

Please check the .m file for the Matlab implementation of the MPAV method.

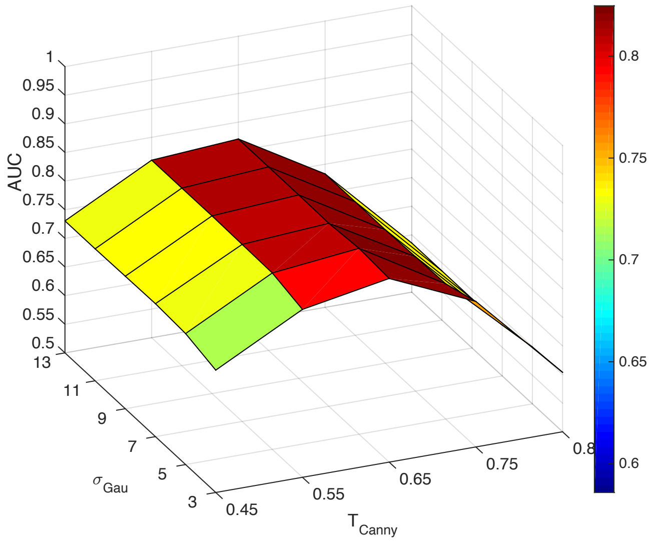

Figure 1: Plot of AUC as a function of different parameters (σ_{Gau} and T_{Canny}) are shown in a 3D plot. $\sigma_{Gau} \in$ [3,5,7,9,11,13] and $T_{Canny} \in$ [0.45, 0.55,0.65,0.75,0.85] form the horizontal axes, the AUC is shown on the vertical axis. A high value AUC is shown as red, whereas a low value AUC is shown as green/blue. One may observe that the performance is less affected by the Gaussian smoothing operation. The MPAV technique achieves max AUC when T_{Canny} =0.65. And the AUC continues to be consistently high when T_{Canny} is in the range of 0.55 to 0.75.

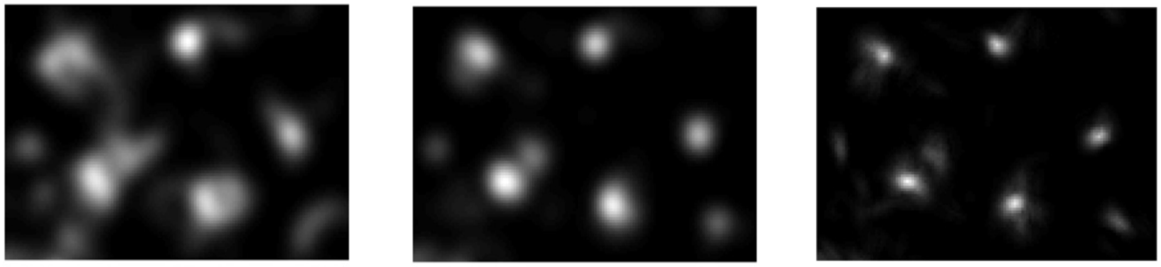

(a) (b) (c)

Figure 2: V_N with different voting angles at each iteration. Setting N=3 and $\Delta \in (\frac{\pi}{4}, \frac{\pi}{7}, \frac{\pi}{28})$, (a) V_1 where $\sigma_1 = \frac{\pi}{4}$, (b) V_2 where $\sigma_2 = \frac{\pi}{7}$, (c) V_3 where $\sigma_3 = \frac{\pi}{28}$. One may observe that as the σ decrease, the V_N has smaller high vote regions which implies more precise identification of nuclear centroids.

S3: MATLAB implementation of the MPAV algorithm.

```
%
% Input
%     -IM:  input image
%     -Para: struct variable for parameters
% Output
%     -im_Vote_Final: the final voting result
%     -im_Vote_Acc: the accumulative map for each iteration
%     -Allim_Vote: record all voting result map at each iteration
%     -bw: the bw indicate the valid region (actually edge) that will
%          contribut for the voting
%     -num_VotingPts?? number of voting pixels that have contribution
% Program written by Cheng LU
% Case Western Reserve University, email:hacylu@gmail.com
% 2016 March 24th

% This function implement a version of the method proposed in manuscript
% "Cheng Lu, et al., Multi-Pass Adaptive Voting for Nuclei Detection in
% Histopathological Images, Nature Scientific Report, 2016"

% example here:
% Para.VotingGap=1;
% Para.rmin=1;  % determine the voting center
% Para.rmax=35;
% Para.theta=pi/4; % the angle of cone-shape
% Para.Factor_Quantize=40; % the higer more accurate
% Para.Sigma=4;
% Para.debug=0;
% Para.ConeshapeRestrict=0;
% Para.N=4; % voting iterations
```

```
% Para.Gaussian_sigma=4;
% Para.CannyEdgeThreshold=0.55;
% theta_min=pi/30;
% Para.thetaSet=[theta_min:(Para.theta-theta_min)/(Para.N-1):Para.theta];
% Para.ObjColor='Black';
% % Para.ObjColor='white';
% Para.Edgemap=[];
% Para.T_PixelNuminComp=50;
% Para.Preprocess_Method='GaussianSmooth_RemoveClosed';
% Para.show=0;
% Para.RetifyBadGradient=1;
% %%% MPAV with 'set to Opposite' stategy
% Para.RetifyGradient_Method='SetToOpposite';
% IM=imread('small_im.tiff');
% [im_Vote_Final_0,im_Vote_Acc_0,Allim_Vote_0,bw_0,num_VotingPts_0]= MPAV(IM(:,:,1),Para);
% figure; imashow(im_Vote_Final_0);

function [im_Vote_Final,im_Vote_Acc,Allim_Vote,bw,num_VotingPts]= MPAV(IM,Para)
%% retify the gradient map
Para.Method=Para.Preprocess_Method;
if Para.RetifyBadGradient
   [Gx,Gy,~,bw,bw_CloseEdges]=LgenerateRetifiedGradientMap4Voting(IM,Para);
else
   [Gx,Gy,~,bw,bw_CloseEdges]=LpreprocessIM4MPV(IM,Para);
end

Gx(~bw)=0;Gy(~bw)=0;
% show(IM,52);hold on;
% quiver(-Gx,-Gy,5,'y');
% hold off;
%show(bw_CloseEdges);
```

```
num_VotingPts=numel(find(Gx~=0));
%% voting parameters setting
VotingGap=Para.VotingGap; % we don't need all pts for the voting, this parameter defineds the sampling gap
r_min = Para.rmin;
r_max = Para.rmax;
IterationN=Para.N;
% the angle of cone-shape
% delta = pi/6;
theta=Para.theta;
% Gaussian Variance
Sigma = Para.Sigma;
bw_edge=bw;
if ~isempty(Para.Edgemap)
   Gx(~Para.Edgemap)=0;
   Gy(~Para.Edgemap)=0;
end

Gmag = sqrt(Gx.^2+Gy.^2);
%% change all the gradient direction to its opposit if object is darker than background
if strcmp(Para.ObjColor,'Black')
   Gx=-Gx;
   Gy=-Gy;
end
%% compute the number of connected components
[m,n] = size(bw_edge);
c1 = bwconncomp(bw_edge);
Allim_Vote=zeros(m,n,IterationN);

%% compute the  patterns
% cal center of image
cy=round(m/2);
```

```
cx=round(n/2);
% compute shift Gaussian kernel center
Factor_Quantize=Para.Factor_Quantize;
res=2*pi/Factor_Quantize;
cos_theta=cos(res:res:2*pi);
sin_theta=sin(res:res:2*pi);

ux = cx+(r_max+r_min)*(cos_theta)/2;
uy = cy+(r_max+r_min)*(sin_theta)/2;



%%% get Gaussian kernel in the valid region for each pattern
imsize=[m n];
ptsIdx_ValidArea=cell(1,Factor_Quantize);% store pts idx list in valiarea
ptsX_ValidArea=cell(1,Factor_Quantize);
ptsY_ValidArea=cell(1,Factor_Quantize);
IntList_ValidArea=cell(1,Factor_Quantize); % store intensity value that corresponds to the pts idx list in
valiarea (ptsIdx_ValidArea)
bw_valid=cell(1,Factor_Quantize);
map_Gaussian_Valid=cell(1,Factor_Quantize);

[px,py] = meshgrid(1:imsize(2),1:imsize(1));

for i=1:Factor_Quantize
    map_gau=zeros(imsize(1),imsize(2));

    [ptsIdx_ValidArea{i},bw_valid{i}]=LgetConeShapeVotingArea(cx,cy,ux(i),uy(i),r_max,r_min,theta,imsize);
    [ptsY_ValidArea{i}, ptsX_ValidArea{i}]=ind2sub(imsize,ptsIdx_ValidArea{i});

    gau = 1/(sqrt(2*pi)*Sigma).*exp(-(sum(bsxfun(@minus,[px(:),py(:)],[ux(i),uy(i)]).^2,2))./(2*Sigma^2));
```

```
    IntList_ValidArea{i}=gau(ptsIdx_ValidArea{i});
    map_gau(ptsIdx_ValidArea{i})=IntList_ValidArea{i};

    map_Gaussian_Valid{i}=map_gau;

end
%% for the first iteration (faster method)
iteration=IterationN;

% accumulated map for recording the voting at each iteration, the V in the paper
map_Acc=zeros(size(bw_edge));
% numVotingPts=1; %ind for the pts involved in voting
theta=Para.thetaSet(iteration);

% gather all pixels that will go for the voting
numAssumeVotPts=80;
AllPixelList=zeros(c1.NumObjects*numAssumeVotPts,1); % preassume that there are 80 at average for each component
idxTemp=1;
for i=1:c1.NumObjects
    AllPixelList(idxTemp:idxTemp+length(c1.PixelIdxList{i})-1)=c1.PixelIdxList{i};
    idxTemp=idxTemp+length(c1.PixelIdxList{i});
end
if c1.NumObjects*numAssumeVotPts>idxTemp-1
    AllPixelList(AllPixelList==0)=[];
end

%%% exclude the pixel whose gradient is equal to 0
AllPixelList(Gmag(AllPixelList)==0)=[];
cos_theta1 =  Gx(AllPixelList)./Gmag(AllPixelList);
sin_theta1 =  Gy(AllPixelList)./Gmag(AllPixelList);
```

```
% compute voting pt x,y location

[cy1,cx1]=ind2sub ([m,n],AllPixelList);

ux1 = cx1+(r_max+r_min).*(cos_theta1)/2;

uy1 = cy1+(r_max+r_min).*(sin_theta1)/2;


%%% mapping all the voting pts' vector to the pre-calculated pattern

matrix_Mapping_degreeDiff=acosd((bsxfun(@times,ux1-cx1,ux-cx)+bsxfun(@times,uy1-cy1,uy-
cy))./(bsxfun(@times,sqrt((ux1-cx1).^2+(uy1-cy1).^2),sqrt((ux-cx).^2+(uy-cy).^2))));

[~,PatternIdx]=min(abs(matrix_Mapping_degreeDiff),[],2);


%     matrix_Mapping_sin=bsxfun(@minus,sin_theta1,sin_theta);

%     matrix_Mapping_cos=bsxfun(@minus,cos_theta1,cos_theta);

%     [~,PatternIdx]=min(abs(matrix_Mapping_sin)+abs(matrix_Mapping_cos),[],2);


%     temp=PatternIdx2-PatternIdx;

%     unique(PatternIdx);


for j = 1:VotingGap:length(AllPixelList)

    map_gau=zeros(size(bw_edge));

    %     fprintf('On the %d/%d voting pixel\n',j,length(AllPixelList));

    i=PatternIdx(j);

    IntList_ValidArea1_temp=IntList_ValidArea{i};

    ptsX_ValidArea1_temp= ptsX_ValidArea{i}+(cx1(j)-cx);

    ptsY_ValidArea1_temp =ptsY_ValidArea{i}+(cy1(j)-cy);


    %%%%%%% ignore pixels out of image range %%%%%%%%%%%%%%%%%%%%%%%%%%

    % if you read this a second time maybe confused, but it is actually due

    % to the 'accompny effect' to remove a pair of pts

    ptsY_ValidArea1_temp(ptsX_ValidArea1_temp<=0)=[];
IntList_ValidArea1_temp(ptsX_ValidArea1_temp<=0)=[];

    ptsX_ValidArea1_temp(ptsX_ValidArea1_temp<=0)=[];
```

```
    ptsX_ValidArea1_temp(ptsY_ValidArea1_temp<=0)=[];
IntList_ValidArea1_temp(ptsY_ValidArea1_temp<=0)=[];

    ptsY_ValidArea1_temp(ptsY_ValidArea1_temp<=0)=[];



ptsY_ValidArea1_temp(ptsX_ValidArea1_temp>imsize(2))=[];IntList_ValidArea1_temp(ptsX_ValidArea1_tem
p>imsize(2))=[];

    ptsX_ValidArea1_temp(ptsX_ValidArea1_temp>imsize(2))=[];



ptsX_ValidArea1_temp(ptsY_ValidArea1_temp>imsize(1))=[];IntList_ValidArea1_temp(ptsY_ValidArea1_temp
>imsize(1))=[];

    ptsY_ValidArea1_temp(ptsY_ValidArea1_temp>imsize(1))=[];

    %%%%%%%%%%%%%%%%%%%%%%%%%%%%%%%%%%%%%%%%%%%%%%%%%%%%%%%%%%
%%%%%%%%%%


    ptsIdx_ValidArea1=sub2ind(imsize,ptsY_ValidArea1_temp,ptsX_ValidArea1_temp);

    map_gau(ptsIdx_ValidArea1)=IntList_ValidArea1_temp;


    map_Acc=map_Acc+Gmag(AllPixelList(j)).*map_gau;

end

Allim_Vote(:,:,iteration)=map_Acc;

%% for the rest iterations

% Para.debug=1;

map_Acc_old=map_Acc;

for iteration=IterationN-1:-1:1 % control the iteration


    % accumulated map for recording the voting at each iteration, the V in the paper

    map_Acc=zeros(size(bw_edge));

    %     numVotingPts=1; %ind for the pts involved in voting

    theta=Para.thetaSet(iteration);


    %%% compute the pattern on the current iteration
```

```
%%%% get Gaussian kernel in the valid region for each pattern
ptsIdx_ValidArea=cell(1,Factor_Quantize);% store pts idx list in valiarea
ptsX_ValidArea=cell(1,Factor_Quantize);
ptsY_ValidArea=cell(1,Factor_Quantize);
IntList_ValidArea=cell(1,Factor_Quantize); % store intensity value that corresponds to the pts idx list in valiarea (ptsIdx_ValidArea)
bw_valid=cell(1,Factor_Quantize);
map_Gaussian_Valid=cell(1,Factor_Quantize);
[px,py] = meshgrid(1:imsize(2),1:imsize(1));

for i=1:Factor_Quantize
    map_gau=zeros(imsize(1),imsize(2));

    [ptsIdx_ValidArea{i},bw_valid{i}]=LgetConeShapeVotingArea(cx,cy,ux(i),uy(i),r_max,r_min,theta,imsize);
    [ptsY_ValidArea{i}, ptsX_ValidArea{i}]=ind2sub(imsize,ptsIdx_ValidArea{i});

    gau = 1/(sqrt(2*pi)*Sigma).*exp(-(sum(bsxfun(@minus,[px(:),py(:)],[ux(i),uy(i)]).^2,2))./(2*Sigma^2));

    IntList_ValidArea{i}=gau(ptsIdx_ValidArea{i});
    map_gau(ptsIdx_ValidArea{i})=IntList_ValidArea{i};

    %    map_gau=reshape(gau,imsize(1),imsize(2)); %show(map_gau);
    %    map_gau(~bw_valid{i})=0;
    map_Gaussian_Valid{i}=map_gau;
    %       show(bw_valid{i},2);
    %    pause();
    %    show(map_Gaussian_Valid{i},3);
end

%%% update the voting direction based on the maximun pixel in
%%% pre-voting area of pre-voting map
```

```
    for j = 1:VotingGap:length(AllPixelList)
        %       fprintf('On the %d/%d voting pixel\n',j,length(AllPixelList));
        map_gau=zeros(size(bw_edge));
        i=PatternIdx(j);
        ptsX_ValidArea1_temp= ptsX_ValidArea{i}+(cx1(j)-cx);
        ptsY_ValidArea1_temp =ptsY_ValidArea{i}+(cy1(j)-cy);

        %%%%%%%% ignore pixels out of image range %%%%%%%%%%%%%%%%%%%%%%%%%%%%%%
        % if you read this a second time maybe confused, but it is actually due
        % to the 'accompny effect' to remove a pair of pts
        ptsY_ValidArea1_temp(ptsX_ValidArea1_temp<=0)=[];
        ptsX_ValidArea1_temp(ptsX_ValidArea1_temp<=0)=[];

        ptsX_ValidArea1_temp(ptsY_ValidArea1_temp<=0)=[];
        ptsY_ValidArea1_temp(ptsY_ValidArea1_temp<=0)=[];

        ptsY_ValidArea1_temp(ptsX_ValidArea1_temp>imsize(2))=[];
        ptsX_ValidArea1_temp(ptsX_ValidArea1_temp>imsize(2))=[];

        ptsX_ValidArea1_temp(ptsY_ValidArea1_temp>imsize(1))=[];
        ptsY_ValidArea1_temp(ptsY_ValidArea1_temp>imsize(1))=[];
        %%%%%%%%%%%%%%%%%%%%%%%%%%%%%%%%%%%%%%%%%%%%%%%%%%%%%%%%%%%%%%%%%%%%%%%
%%%%%%%%%%%%
        if isempty(ptsY_ValidArea1_temp)
            continue;
        end
        ptsIdx_ValidArea1=sub2ind(imsize,ptsY_ValidArea1_temp,ptsX_ValidArea1_temp);
        [~,maxIdx]=max(map_Acc_old(ptsIdx_ValidArea1));
        maxIdxinIM=ptsIdx_ValidArea1(maxIdx(1));
        [max_r,max_c]=ind2sub([m,n],maxIdxinIM);
```

```
        if Para.debug
            bw_valid=zeros(m,n);bw_valid(ptsIdx_ValidArea1)=1;
            LshowBWonIM(bw_valid,map_Acc_old,109);hold on;
            plot(max_c,max_r,'*r');
            plot(cx1(j),cy1(j),'bo');
            hold off;
        end

        dx=max_c-cx1(j);dy=max_r-cy1(j);
        cos_theta_temp=dx/sqrt(dx^2+dy^2);
        sin_theta_temp=dy/sqrt(dx^2+dy^2);

        matrix_Mapping_sin=bsxfun(@minus,sin_theta_temp,sin_theta);
        matrix_Mapping_cos=bsxfun(@minus,cos_theta_temp,cos_theta);
        [~,PatternIdx(j)]=min(abs(matrix_Mapping_sin)+abs(matrix_Mapping_cos),[],2);

        i=PatternIdx(j);
        IntList_ValidArea1_temp=IntList_ValidArea{i};
        ptsX_ValidArea1_temp= ptsX_ValidArea{i}+(cx1(j)-cx);
        ptsY_ValidArea1_temp =ptsY_ValidArea{i}+(cy1(j)-cy);

        %%%%%%%% ignore pixels out of image range %%%%%%%%%%%%%%%%%%%%%%%%%%%%%
        % if you read this a second time maybe confused, but it is actually due
        % to the 'accompny effect' to remove a pair of pts
        ptsY_ValidArea1_temp(ptsX_ValidArea1_temp<=0)=[];
IntList_ValidArea1_temp(ptsX_ValidArea1_temp<=0)=[];
        ptsX_ValidArea1_temp(ptsX_ValidArea1_temp<=0)=[];

        ptsX_ValidArea1_temp(ptsY_ValidArea1_temp<=0)=[];
IntList_ValidArea1_temp(ptsY_ValidArea1_temp<=0)=[];
        ptsY_ValidArea1_temp(ptsY_ValidArea1_temp<=0)=[];
```

```
ptsY_ValidArea1_temp(ptsX_ValidArea1_temp>imsize(2))=[];IntList_ValidArea1_temp(ptsX_ValidArea1_tem
p>imsize(2))=[];
        ptsX_ValidArea1_temp(ptsX_ValidArea1_temp>imsize(2))=[];


ptsX_ValidArea1_temp(ptsY_ValidArea1_temp>imsize(1))=[];IntList_ValidArea1_temp(ptsY_ValidArea1_temp
>imsize(1))=[];
        ptsY_ValidArea1_temp(ptsY_ValidArea1_temp>imsize(1))=[];
        %%%%%%%%%%%%%%%%%%%%%%%%%%%%%%%%%%%%%%%%%%%%%%%%%%%%%%%%%%%%
%%%%%%%%%%%

        ptsIdx_ValidArea1=sub2ind(imsize,ptsY_ValidArea1_temp,ptsX_ValidArea1_temp);
        map_gau(ptsIdx_ValidArea1)=IntList_ValidArea1_temp;

        if Para.debug
            bw_valid=zeros(m,n);bw_valid(ptsIdx_ValidArea1)=1;
            LshowBWonIM(bw_valid,map_gau,110);hold on;
            plot(max_c,max_r,'*r');
            plot(cx1(j),cy1(j),'bo');
            hold off;
        end
        map_Acc=map_Acc+Gmag(AllPixelList(j)).*map_gau;
    end
    %     show(map_Acc);
    Allim_Vote(:,:,iteration)=map_Acc;
    map_Acc_old=map_Acc;
end
im_Vote_Acc=sum(Allim_Vote,3);
im_Vote_Final=Allim_Vote(:,:,1);

%% add the closed edge map on to the voting map
% im_Vote_Final=zeros(size(IM,1),size(IM,2));
```

```
maxV=max(im_Vote_Final(:));
s=regionprops(bw_CloseEdges,'Centroid');
temp=[s.Centroid];
x=round(temp(1:2:end));y=round(temp(2:2:end));
idx=sub2ind([size(IM,1),size(IM,2)],y,x);

im_Vote_Final(idx)=maxV;
% show(im_Vote_Final);
% LshowCrossfromBWonIM(im_Vote_Final>100,IM,3,'MPAV-GRS2');
end



function [Gx,Gy,IM_ws,bw,bw_CloseEdges]=LgenerateRetifiedGradientMap4Voting(IM,Para)

T_PixelNOinComp=Para.T_PixelNuminComp; % control the edge fragment that go for voting

Para.Method=Para.Preprocess_Method;
[Gx,Gy,IM_ws,bw,bw_CloseEdges]=LpreprocessIM4MPV(IM,Para);

px=Gx;
py=Gy;
% show(bw);
cc=bwconncomp(bw);
ss=regionprops(cc,'ConvexHull','PixelIdxList','Centroid');
for i=1:cc.NumObjects
  curPixelsList=ss(i).PixelIdxList;
  numPixels=length(curPixelsList);
  if numPixels>T_PixelNOinComp
    curHull=ss(i).ConvexHull;
    bw_curEdge=zeros(size(bw));
    bw_curEdge(curPixelsList)=1;
```

```
bw_curHull=poly2mask(curHull(:,1),curHull(:,2),size(bw,1),size(bw,2));

cc_hull=bwconncomp(bw_curHull);
ss_hull=regionprops(cc_hull,'Centroid');
curHullCentroid=ss_hull.Centroid;
if Para.debug
  % show the graident info and centroid within the hull
  show(bw_curHull,14); hold on;
  pxshown=px; pyshown=py;
  pxshown(~bw_curEdge)=0;pyshown(~bw_curEdge)=0;
  quiver(-pxshown,-pyshown,5,'g');
  plot(curHullCentroid(1),curHullCentroid(2),'rp','MarkerSize',20);
  hold off;

  show(bw_curEdge,15); hold on;
  pxshown=px; pyshown=py;
  pxshown(~bw_curEdge)=0;pyshown(~bw_curEdge)=0;
  quiver(-pxshown,-pyshown,5,'g');
  hold off;
end
% for each pixel with valid gradient(i.e., G>0), we retify its gradient direction
% debug version here
AllBadG=[]; % record gradient locations
AllGoodG=[];

for j=1:numPixels
  [curP_y,curP_x]=ind2sub(size(bw),curPixelsList(j));
  if Para.debug
    %          show(bw_curHull,14); hold on;
    %          pxshown=px; pyshown=py;
```

```
    %               pxshown(~bw_curEdge)=0;pyshown(~bw_curEdge)=0;
    %               quiver(-pxshown,-pyshown,5,'b');
    %               plot(curHullCentroid(1),curHullCentroid(2),'rp','MarkerSize',20);
    %               plot(curP_x,curP_y,'rs');
    %               hold off;
  end
  curPG_x=px(curPixelsList(j));curPG_y=py(curPixelsList(j));

  %          vector_PC=curHullCentroid-[curP_x,curP_y]; % this one should
  %          be correct,why?
  vector_PC=[curP_x,curP_y]-curHullCentroid;
  angle=acos(sum(vector_PC.*[curPG_x,curPG_y])/(norm(vector_PC)*norm([curPG_x,curPG_y])));

  % pick gradient based on angle
  if ~(0<angle&&angle<pi/2)
    %record for latter use
    AllBadG=[AllBadG; curP_x,curP_y];

  else
    AllGoodG=[AllGoodG; curP_x,curP_y];
  end
end

if Para.debug
  show(bw_curHull,14); hold on;
  pxshown=px; pyshown=py;

  pxshown(~bw_curEdge)=0;pyshown(~bw_curEdge)=0;
  quiver(-pxshown,-pyshown,5,'b');
  plot(curHullCentroid(1),curHullCentroid(2),'ro','MarkerSize',20);
  if ~isempty(AllBadG)
```

```
        plot(AllBadG(:,1),AllBadG(:,2),'ks');
    end
    if ~isempty(AllGoodG)
        plot(AllGoodG(:,1),AllGoodG(:,2),'rp','MarkerSize',10);
        hold off;
    end
end
%% deal with the bad gradient, we set them to 0 here, we can have better choice
%%% lulu
if strcmp(Para.RetifyGradient_Method,'SetTo0')
    if size(AllBadG,1)>0
        AllBadG_List=sub2ind(size(bw),AllBadG(:,2),AllBadG(:,1));
        Gx(AllBadG_List)=0;
        Gy(AllBadG_List)=0;
    end
end

if strcmp(Para.RetifyGradient_Method,'SetToOpposite')
    if size(AllBadG,1)>0
        AllBadG_List=sub2ind(size(bw),AllBadG(:,2),AllBadG(:,1));
        Gx(AllBadG_List)=-Gx(AllBadG_List);
        Gy(AllBadG_List)=-Gy(AllBadG_List);
    end
end

if Para.debug
    show(bw_curHull,17); hold on;
    pxshown=Gx; pyshown=Gy;

    pxshown(~bw_curEdge)=0;pyshown(~bw_curEdge)=0;
    quiver(-pxshown,-pyshown,5,'b');
```

```
            plot(curHullCentroid(1),curHullCentroid(2),'ro','MarkerSize',20);
            if ~isempty(AllBadG)
                plot(AllBadG(:,1),AllBadG(:,2),'ks');
            end
            if ~isempty(AllGoodG)
                plot(AllGoodG(:,1),AllGoodG(:,2),'rp','MarkerSize',10);
                hold off;
            end
        end
    end
end
end

function [Gx,Gy,IM_ws,bw,bw_CloseEdges]=LpreprocessIM4MPV(IM,Para)
if isempty(Para.Gaussian_sigma)
    Para.Gaussian_sigma=5;
end

%% M-------- GaussianSmooth_Hysthersh
if strcmp(Para.Method,'GaussianSmooth_Hysthersh')
    % smooth image
    IM_ws=imfilter(double(IM),fspecial('Gaussian',[5 5],1),'same','conv','replicate');

    [Gx,Gy] = gradient(IM_ws);% gradiet maps that will return

    % let the edges connecting
    bw = hysthresh(255-IM_ws, 70, 100);
end
%% M-------- GaussianSmooth_Hysthersh
if strcmp(Para.Method,'GaussianSmooth_RemoveClosed')
    % smooth image
```

```
    IM_ws=imfilter(double(IM),fspecial('Gaussian',[Para.Gaussian_sigma
Para.Gaussian_sigma],1),'same','conv','replicate');
    %    show(IM_ws);
    [Gx,Gy] = gradient(IM_ws);% gradiet maps that will return

    %% for closed edges, we treat them as nuclei boundary and pick them out first
    %    CannyEdgeThreshold=0.55;% higher, less edges
    CannyEdgeThreshold=Para.CannyEdgeThreshold;

    IM_edge=edge(IM_ws, 'canny',CannyEdgeThreshold);
    %    show(IM_edge);
    IM_fill=imfill(IM_edge,'holes');
    %    show(IM_fill);
    IM_temp=IM_fill&~IM_edge;
    %     show(IM_temp);
    IM_temp_d=imreconstruct(IM_temp,IM_fill);
    bw_CloseEdges=LgetValidNucleifromClosedEdges(IM_temp_d,0.65);
    %     show(bw_CloseEdges);

    IM_edge_left= IM_edge&~bw_CloseEdges; % show(IM_edge_left)
    if Para.show
        LshowTwoKindofCountouronIM(bw_CloseEdges, IM_edge_left,IM_ws,6);
    end
    %     bw=IM_edge_left;
    %% remove the long edge fragments, since they are definitely the noise fragments
    bw=LremoveHighAxisRatio(IM_edge_left,5);
end
%% M--------
if strcmp(Para.Method,'Wiener_RemoveNoise')
    %%
```

```
CannyEdgeThreshold=Para.CannyEdgeThreshold;

Para_wiener=[4 4];
temp_wien = wiener2(IM,Para_wiener);
%    show(temp_wien,2)
IM_ws=temp_wien;
temp_edge=edge(temp_wien, 'canny',CannyEdgeThreshold);


% [px,py] = gradient(imfilter(double(temp_wien),fspecial('Gaussian',[5 5],1),'same','conv','replicate'));
[px,py] = gradient(double(temp_wien));

bw_edge=temp_edge;
px(~bw_edge)=0;py(~bw_edge)=0;

if Para.show
   show(bw_edge,53);hold on;
   quiver(-px,-py,5,'b');
   hold off;
end
%% for closed edges, we treat them as nuclei boundary and pick them out first

temp_wien = wiener2(IM,Para_wiener);
% show(temp_wien,2)
IM_edge=edge(temp_wien, 'canny',CannyEdgeThreshold);

IM_fill=imfill(IM_edge,'holes');
%    show(IM_fill);
IM_temp=IM_fill&~IM_edge;
%     show(IM_temp);
IM_temp_d=imreconstruct(IM_temp,IM_fill);
```

```
    bw_CloseEdges=LgetValidNucleifromClosedEdges(IM_temp_d,0.65);



    IM_edge_left= IM_edge&~bw_CloseEdges; % show(IM_edge)
    if Para.show
        LshowTwoKindofCountouronIM(bw_CloseEdges, IM_edge_left,temp_wien,6);
    end
    %% remove the narrow and long edge, since they are not likely the contour of nuclei region

    IM_edge_left_nl=LremoveHighAxisRatio(IM_edge_left,6,1);
    if Para.show
        LshowTwoKindofCountouronIM(IM_edge_left, IM_edge_left_nl,temp_wien,7);
    end
    bw=IM_edge_left_nl;

    [Gx,Gy] = gradient(double(IM_ws));

end
end



% cal the cone shape voting area
% x, y represent the current piont
% ux, uy represent the shift Gaussian center piont
% r_max, r_min represent the maximum and minimum range of the cone
% theta is the one side anlge allowed for the cone
% imsize is the size of image
% this function returns the pixel list that are located in the valie cone
% shape area for the voting
function [ptsIdx_ValidArea,bw_valid]=LgetConeShapeVotingArea(x,y,ux,uy,r_max,r_min,theta,imsize)
%% get ring first
```

```
% bw=zeros(imsize);
% bw=(x-ux).^2+(y-uy).^2<r_max;

[px,py] = meshgrid(1:imsize(2),1:imsize(1));

bw1=((px-x).^2+(py-y).^2)<r_max^2;
% show(bw1);
bw2=((px-x).^2+(py-y).^2)<r_min^2;
% show(bw2);

bw_ring=bw1&~bw2;
% show(bw_ring);

%% get beam
alpha=atan((uy-y)/(ux-x));
% current Graident voting  Angle is denoted as alpha
% alpha=atan((uy-y)/(ux-x));
% cal k for two lines
k1=tan(alpha-theta);
k2=tan(alpha+theta);
b1=y-k1*x;
b2=y-k2*x;

bw3=k1*px-py+b1<0;
% if isnan(uy)
%     pause();
% end
if bw3(floor(uy),floor(ux))~=true
   bw3=k1*px-py+b1>0;
end
%show(bw3);
```

```
bw4=k2*px-py+b2<0;
if bw4(floor(uy),floor(ux))~=true
   bw4=k2*px-py+b2>0;
end
bw_beam=bw3& bw4;
%% get cone shape valid area
bw_valid=bw_beam&bw_ring;
%%
ptsIdx_ValidArea=find(bw_valid(:)>0);
end

function bwValid=LgetValidNucleifromClosedEdges(bw,ratio)
cc=bwconncomp(bw);
stats  = regionprops(cc, 'Extent');
idx = find([stats.Extent] > ratio);
bwValid = ismember(labelmatrix(cc), idx);
end

% edgeMap: the binary map in which 1 represent the edge, otherwise
% background
% TRatio: the ratio of major / minor axis
function edgeMap=LremoveHighAxisRatio(edgeMap,TRatio)
cc= bwconncomp(edgeMap);
stats = regionprops(cc,'MajorAxisLength','MinorAxisLength');
idx = find([stats.MajorAxisLength]./[stats.MinorAxisLength] < TRatio);
edgeMap= ismember(labelmatrix(cc), idx);
end
```
